# Challenges of Loss to Follow-up in Tuberculosis Research

**DOI:** 10.1371/journal.pone.0040183

**Published:** 2012-07-12

**Authors:** Thomas N. Nissen, Michala V. Rose, Godfather Kimaro, Ib C. Bygbjerg, Sayoki G. Mfinanga, Pernille Ravn

**Affiliations:** 1 Clinical Research Unit, Hvidovre Hospital, Hvidovre, Denmark; 2 Department of International Health, Immunology and Microbiology, Copenhagen University, Copenhagen, Denmark; 3 National Institute for Medical Research Muhimbili Centre, Dar es Salaam, United Republic of Tanzania; 4 Department of Infectious Diseases, University Hospital Hillerød, Hillerød, Denmark; Menzies School of Health Research, Australia

## Abstract

**Background:**

In studies evaluating methods for diagnosing tuberculosis (TB), follow-up to verify the presence or absence of active TB is crucial and high dropout rates may significantly affect the validity of the results. In a study assessing the diagnostic performance of the QuantiFERON®-TB Gold In-Tube test in TB suspect children in Tanzania, factors influencing patient adherence to attend follow-up examinations and reasons for not attending were examined.

**Methods:**

In 160 children who attended and 102 children who did not attend scheduled 2-month follow-up baseline health characteristics, demographic data and risk factors for not attending follow-up were determined. Qualitative interviews were used to understand patient and caretakers reasons for not returning for scheduled follow-up.

**Results:**

Being treated for active TB in the DOTS program (OR: 4.14; 95% CI:1.99–8.62;p-value<0.001) and receiving money for the bus fare (OR:129; 95% CI 16->100;P-value<0.001) were positive predictors for attending follow-up at 2 months, and 21/85(25%) of children not attending scheduled follow-up had died. Interviews revealed that limited financial resources, i.e. lack of money for transportation and poor communication, were related to non-adherence.

**Conclusion:**

Patients lost to follow-up is a potential problem for TB research. Receiving money for transportation to the hospital and communication is crucial for adherence to follow-up conducted at a study facility. Strategies to ensure follow-up should be part of any study protocol.

## Introduction

Poor follow-up adherence may lead to increased cost of research projects, underpowered studies, biased results, and in worst case incorrect conclusions because of missing data [1]. Missing data is pervasive in clinical trials and has a major impact on the quality of research. Hollis and Campbell reviewed 249 clinical trials in 1997 and found that 75% of the studies were affected by missing data related to primary outcome variables [2]. Several studies from East Africa have reported that patient adherence to follow-up is a significant problem when analysing data [3], [4]. Inadequate adherence in research studies may necessitate increasing the sample size in order to evaluate the research hypotheses, which lengthens investigations and requires more staff and money.

Most published research about follow-up adherence focuses on adherence to treatment whilst little is published on follow-up adherence in a research situation. Clarifying risk factors for poor adherence, in relation to attending scheduled follow-up, will be useful for researchers in general, when planning a clinical trial.

In a study evaluating new diagnostic tools for childhood TB in Tanga region, Tanzania, (IMPACT-TB) patients who did not return, for the scheduled 2-month follow-up evaluation, were routinely actively traced in the villages where follow-up was then conducted and caretakers interviewed.

The aim of this study was to identify factors influencing patient adherence to attend scheduled follow-up and reasons for not attending using baseline demographic measures and health characteristics as well as qualitative interviews with caretakers and staff members.

## Materials and Methods

### Study Population

The IMPACT-TB study assessing the diagnostic performance of the QuantiFERON®-TB Gold In-Tube test in TB suspect children was conducted in Muheza District, Tanzania, a high TB burden, poor rural district with a population of 209.480, where 90% are engaged in peasantry, fishery and small-scale business. The study included children <15 years presenting with signs and symptoms of TB at the hospital paediatric ward and TB and HIV clinics [5].

### Follow up Procedures

The children were assessed for TB and then scheduled for follow-up evaluation at 2 and 6 months after inclusion, in order to confirm the initial discharge diagnosis of either “confirmed TB” or “not-TB”. Follow-up was essential to ensure correct TB diagnosis, and caretakers received money for the bus fare in order to return for follow-up.

Actively tracing children, who did not turn up for the scheduled 2-month follow-up, by visiting their households, was a part of the overall IMPACT-TB study design. Clinical information concerning the child’s health status was recorded and a clinical examination conducted, using the same procedure for both scheduled follow-up and follow-up of actively traced defaulters.

### Data Collection

We used a method of triangulation where we applied and combined both quantitative and qualitative research methodologies in order to check the mutual consistency of our results and gain a better understanding of the meaning and implications of the findings [6]–[8]. Baseline data on the child’s medical condition and demographic data were obtained from the IMPACT-TB database and used to assess risk factors that may affect follow-up adherence.

Children and their caretakers, as well as staff and local researchers, were interviewed to explore experiences and perceptions of the follow-up evaluation and reasons for attending or not attending follow-up. The patients and caretakers were selected to reflect diverse geographic locations as well as diverse inclusion dates, to broadly reflect the general IMPACT-TB study population. The number of interviews was determined by data saturation, meaning that additional interviews were no longer considered to add any new insights to the collected data [9] Of the 27 interviewed children and caretakers, 17 had not returned for follow-up, and 10 had returned to the study facility as scheduled at discharge. Interviews were conducted by the PI, a village health worker and a clinical officer. The PI interviewed all the patients using the same translator. Afterwards the interviews were transcribed by a third person. Both the Swahili and the English version of the interviews were translated and transcribed. An interview guide using open-ended questions was used and emerging themes and hypotheses from early interviews were explored in subsequent interviews. The aim of the interviews was not to generate new theories, but to elaborate answers to the questions raised by the initial findings of quantitative database analysis of risk factors for not attending follow up such as; clinical and social factors and demographics. In addition we explored perceptions of the IMPACT-TB study and knowledge about tuberculosis.

The qualitative data were analyzed through a four-step procedure [10], [11]: a) getting an overall impression of the data by reading the interviews without making notes, b) identifying meaning units in the second reading of all the material, c) abstracting the contents of individual meaning units, and d) summarising their importance. This was a dynamic process, where the different meaning units were re-evaluated several times. Consequently the identified meaning units were de-contextualized and organized into themes. For each theme the pieces of text were now re-contextualized, explaining and elaborating the themes. Finally the themes were read in the light of the core data to certify the relevance in relation to the original interview material.

### Statistical Methods and Data Analysis

The chi square test was used in the univariable analysis to assess the association between variables suspected of affecting follow-up and adherence to follow-up. A p-value of 0.05 was considered statistically significant. Six factors were considered in the analysis; sex, age, TB diagnosis, HIV status, nutritional status and whether bus fare was received or not. In the multivariable analysis an adjusted odds ratio was calculated using binary logistic regression, adjusting only for the specific factors suspected to affect the variable investigated.

All the statistical analysis were made in SAS 9.1.3 Service Pack 4 WIN_PRO platform.

### Ethical Concerns

This study falls within the scope of the IMPACT-TB study “Improving prevention and diagnosis of active TB in children in North East Tanzania”, approved by Tanzanian Medical Research Coordinating Committee (NIMR/HQ/R.8a/Vol IX/584) and evaluated by the Danish Central Ethical Committee without any objections. All informants in the study gave both oral and written informed consent.

## Results

**Figure 1:**
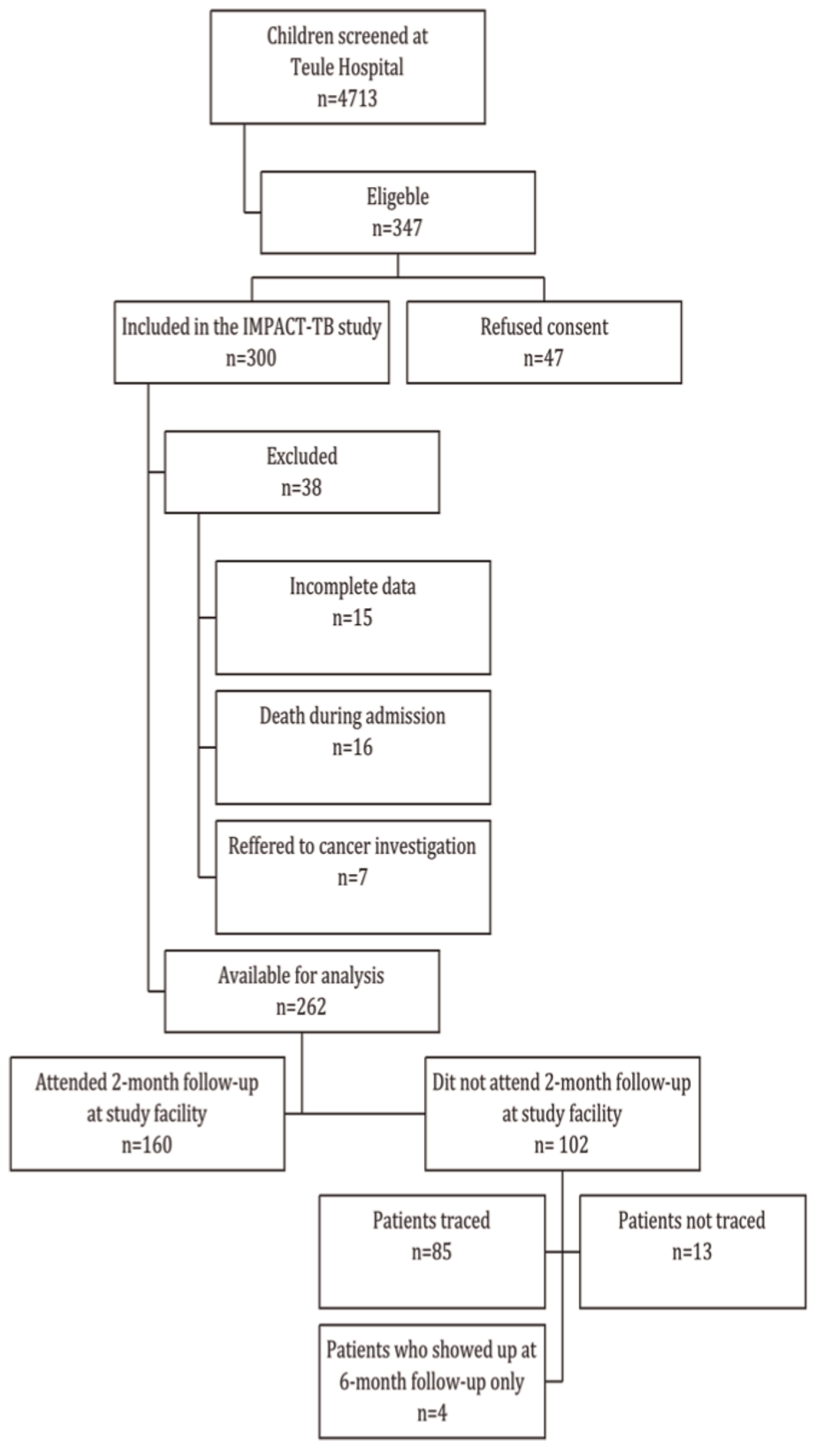
Flowchart of children included in the study.

Between April 2008 and June 2010, a total of 4713 patients were screened for symptoms of TB at Muheza District Hospital. 347 patients were found eligible for the IMPACT-TB study, 47 refused consent and an additional 38 patients were excluded from the analysis of the 2-month follow-up data; 15 with incomplete data, 16 who died during admission at the hospital and 7 that had been referred to Ocean Road Cancer Centre in Dar es Salaam, where follow-up was not possible. We included 262 children in the data analysis. We found that 102/262 (39%) had not attended scheduled 2-month follow-up. Of these 4 were seen at 6 month follow-up and 85 were found during active tracing leaving only 13/262 (5%) completely lost to follow up. **(** **Fig. 1** **)**.

Follow-up adherence at 2 months was affected by the child’s TB diagnosis and whether the caretaker received money for bus fare to return for follow-up; 83.6% (56/67) of the patients diagnosed with TB and receiving treatment compared to 53.3% (104/195) of the patients that were not diagnosed with TB, and therefore not receiving treatment, came for 2-month follow-up (p-value <0.001). At inclusion all caretakers were informed of reimbursement of the bus fare when discharged from the hospital. Some patients managed to leave the hospital without approaching the study facility for the bus fare reimbursement, which strongly affected follow-up adherence: 72.3% (159/220) of patients who received money for the bus fare compared to 2.4% (1/42) of those who did not receive money for the bus fare, came for 2-month follow-up (p-value  =  <0.001). In the multivariable analysis both receiving treatment for TB and receiving money for the bus fare, were positive predictors for attending 2-month follow-up. There was no statistically significant effect of sex, age, HIV status or nutrition status on adherence to follow-up. **(** **Table 1** **)**.

**Table 1:** 

	Characteristics of adherent and non-adherent patients			Factors associated with adherence to follow-up at study facility		
Characteristics	Non-adherent	Adherent	P-value	Adj OR	95%(CI)	P-value
	n = 102 (%)	n = 160 (%)				
**Sex** ^a^						
Male	53(35.3)	97(64.7)	0.17	1.40	(0.84–2.32)	0.20
Female	49(43.8)	63(56.3)		1		–
**Age** ^b^						
<2 years	51(46.0)	60(54.1)	0.13	0.58	(0.32–1.06)	0.07
2–5 years	24(34.8)	45(65.2)		0.91	(0.46–1.78)	0.77
>5 years	27(32.9)	55(67.1)		1		–
**TB status** ^1, c^						
Positive	11(16.4)	56(83.6)	<0.001	4.14	(1.99–8,62)	<0.001
Negative	91(46.7)	104(53.3)		1		–
**HIV status** ^2, d^						
Positive	26(32.9)	53(67.1)	0.21	1.39	(0.79–2.50)	0.25
Negative	74(41.1)	106(58.9)		1		–
**Nutrition status** ^3, e^						
Z-score ^3^ −2	26(38.8)	41(61.2)	0.89	0.97	(0.52–1.81)	0.93
Z-score < −2	76(39.8)	115(60.2)		1		–
**Money for bus fare** ^ 4, f^						
Received	61(27.7)	159(72.3)	<0.001	129	(16−>100)	<0.001
Not received	41(97.6)	1(2.4)		1		-

1,3,4 5 patients were excluded from the OR analysis due to missing values^ 2^3 patients were excluded from the OR analysis due to missing values ^a^OR was adjusted for age ^b^OR was adjusted for sex ^c^OR was adjusted for age, sex, HIV status and nutrition status ^d^OR was adjusted for age and sex ^e^OR was adjusted for age, sex, TB status and HIV status ^f^OR was adjusted for age, sex, HIV status, TB status and nutrition status.

The visits to the households of the defaulters revealed that 25% (21/85) of the children that did not attend scheduled 2-month follow-up, had died. It was not possible to obtain reliable information about the causes of death. Examining the remaining 64 defaulting children who were still alive, we found that 77% of the children were healthy, 8% (5/64) had problems with fever, 23% (15/64) coughing and 9% (6/64) experienced weight loss or failure to gain weight. Eight children had more than one health problem. Caretakers reported that 41% (26/64) of the children had been readmitted, at least once, to a health facility or hospital since inclusion in the study. Four children were found to have signs and symptoms suggestive of TB and were referred to a TB clinic for further investigation. The caretakers were asked about reasons for not attending 2-month follow-up. Death of the child (21/85) was the most common reason, whilst not receiving bus fare was the reason given by 7% (6/85) of the caretakers, and 5% (4/85) were short of money in general ***(*** ***Table 2*** ***)***.

**Table 2:** Reasons given by caretakers for not attending 2-month follow-up at study facility.

Reasons given by caretaker	n = 85(%)
Child died	21 (25%)
Moved away	12 (14%)
Travelling	14 (16%)
Did not get money for bus fare	6 (7%)
Had no money	4 (5%)
Death in family	4 (5%)
Did not know	3 (4%)
Forgot	8 (9%)
Other	14 (16%)

The qualitative interviews, to explore in more detail the reasons for not attending follow-up, revealed four main themes: financial resources, health of the child, communication and domestic factors.

### Financial Limitations

The single most common reason given for not attending 2-month follow-up was lack of money. A caretaker said when interviewed:

*“The reason is just that the situation is hard. Getting money here is a problem. Do you think one can stay with that money up to that date? If I had money I would have taken the child to hospital.”*

The financial problems are sometimes even more complicated, when the caretaker lives alone or is widowed. Then the mother has to spend all her time working, so she can provide food for her children. An interviewed mother put it this way:

*“I am a single mother; I have to work so that I can get bus fare to get there. Then you say that we don’t want to be treated, we want, but transport is a big problem*.”

Some patients did not receive the bus fare money when discharged from the hospital, and argue that this is the reason why they did not attend 2-month follow-up. Other patients are aware that they got money for the bus fare, but admit, that they had used the money:

*“I was given bus fare yes, then I spent the money.”*

Others told us that the amount given could not cover the expenses for transportation. One patient said that the price of fuel had gone up, and the ticket prices were raised:

*“There was a fuel hike and bus fare was raised. That is why I did not go back. I was only given 2000 Tsh and bus fare is 1500 Tsh each way”*

It seems clear that saving money for transport can be a great problem. The bus fare provided by the project appeared to be of great value to the patients, and absence of that reimbursement, removed a great part of the motivation for attending 2-month follow-up. In a resource-limited area where most only have enough money for subsistence, two months is a long time to save money, to spend on the bus fare. As a research project it is important to make sure, that the patients don’t have unnecessary expenses through their participation in the study.

### Health of the Child

The caretakers mentioned the health of their child as a great motivator for attending follow-up. Some caretakers were eager to adhere to the study procedures and knew that they would not be able to pay, for the investigations and clinical tests that were performed, themselves. A caretaker said:

*“What motivated me to take her back is her chest, she is having great problems with the chest, and since they are doing tests, I had to take her because she is really worrying me every now and then because of the chest.”*

Several caretakers had poor trust in their own ability to notice if their child was well or not. The follow-up examination was seen as an opportunity to ensure the good health of their child and get advice on how to best take care of their child.

*“I came here knowing that it’s important to me because I will get to know my child’s health status, how he is faring whether good or bad, or if there is anything that has risen. I have also come for follow-up to check the progress of the child because her condition is still not good.”*

Whether the child was healthy or not, caretakers wanted to bring their children for further investigations. Many of them said, that you can never have your child examined too much, and that you may not otherwise notice if the child is suffering from some disease that you do not know about. In a setting where hospital treatment can be relatively expensive for the patients, it could be very motivating that the clinical investigations of the research study were free of charge.

Many caretakers related the quality of the project to the health of the child, but generally the impression was that caretakers had good faith in the quality of the project. None of the interviewed caretakers could describe the main objectives of the study, but many argued that the main goals were to erase TB and help people.

### Communication

In general, the perception of the communication between the staff and the study participants was good. When discharged, patients were given a discharge card with the 2-month follow-up date and money for the bus fare. Some patients, when discharged in the late hours or during the weekend, expressed difficulties in finding staff from the project, before leaving the hospital. In which case they did not get a date for the 2-month follow-up or money for the bus-fare. One caretaker said:

*“I was discharged on a Saturday and I asked the doctor that discharged me but he told me that they [project staff] don’t come to work on Saturdays.”*

Some patients experienced that they came to the hospital for follow-up, but were sent home by the staff of the project. Reasons giving by the staff were usually that the date for follow-up was overdue or that working hours were finished and the patient had to come another day, as explained by a caretaker:

*“When I came the child was sick, I went there and looked for them but they were not there, I found them at the canteen, they told me that they were leaving at that time.”*

Children who were diagnosed with tuberculosis were referred to the hospital TB clinic for anti-TB treatment. That led to confusion as the caretakers were not aware that they were supposed to go both to the TB clinic for treatment and also to the study facility for follow-up evaluation.

*“I simply didn’t understand. The other day I asked the nurse why they did not give me an appointment date for going back there. I didn’t understand because I went there several times to take medicine.”*

The anti-TB treatment was often continued at local health centres and dispensaries, leading to confusion as the TB clinic staff told patients not to bother coming to the hospital for treatment whilst they had been told to come back for 2 month follow-up by the study team.

*“We went to Teule and we were told to be going to Kabuku (local health facility) for treatment. They had booked him for 18^th^, but when I went there I was told that I should not trouble myself to go that far and that I should be treated just there (Kabuku).”*

Thus communication at all levels is important. When there are several actors in play, (patients, project staff, TB clinic staff) it is very important, that communication is clear and consistent. As a project it is difficult to change locations of hospital clinics, but short distance between the project, the TB clinic and the children’s ward would reduce the possibility of mixing up appointments at the different places. The patients express willingness to attend follow-up, but get confused when told different things at the TB clinic and at the project facility.

### Domestic Factors

Usually it is a female caretaker, who brings the child to the hospital, either the mother, the grandmother, a female sibling or a neighbour. The project staff mentioned several cases, where mothers would hesitate to give informed consent to the study because of worries that the father might dislike the child being included in a medical study. When interviewed in the villages a few caretakers talked about domestic disagreements concerning inclusion in a study. A staff member of the study explained:

*“…others tell us that their husbands don’t like them to come here, and others refuse consent due to the problem, that their husband is not here so they have got no permission to enter the children at the project.”*

When a person other than the primary caretaker admits the child to the hospital, information about treatment and study inclusion is seldom passed on satisfactorily. That leads to confusion and often also defaulting at 2-month follow-up. An interviewed mother explained:

*“The father of the child had the discharge form, and I did not know what date the child was supposed to go back. When he brought the child to hospital, I had given birth to this child here and I was at home.”*

Some villages have a village health worker who ensures that children are taken to hospital when needed. Some children were accompanied by a village health worker, who also ensured that the child was taken back for follow-up. A mother of a child that defaulted told us:

*“As I had told you earlier, that lady who is our village health worker came here and took the child, she told me that she is taking the child for tests, and when the child is required to be taken back for follow-up she comes and take takes her back, she doesn’t tell me when the child is expected back for follow-up.”*

Many caretakers explained that illness or death among other family members affected their ability to attend follow-up on the given date. The primary caretaker sometimes had to take care of other family members, or the family had to travel away to attend a funeral. A mother explained:

*“…after I was discharged my mother was also admitted in a hospital in Dar es Salaam. I had no one to leave my child with, so I left with her.”*

Domestic factors are important. Personal matters and practical problems remove focus from the 2-month follow-up date. It is important to be aware that some female caretakers are not able to give consent on behalf of the child to participate in a study. It is important that caretakers are not encouraged to give consent that their husbands would not agree to. It could be an option to send caretakers home to discuss with their husbands before giving consent, or to encourage the husband to talk to the study staff for further information about the study. It is not possible to prevent illness and death among other family members, but it is important, that the caretakers know what to do, when the 2-month follow-up date is overdue.

## Discussion

We found that 39% of the study participants did not return for their scheduled 2-month follow-up however by visiting households and tracing defaulters we were able to obtain follow-up data for 95% of the study population. Follow-up adherence was influenced by factors such as travel costs and insufficient communication, as well as death after discharge whilst adherence amongst children treated for TB was high. We did not find any association with sex or age. The qualitative interviews revealed four main themes: financial limitations, communication, health of the child, and domestic factors.

In our study the non-adherent rate of 39% was very high and if active tracing of defaulters had not been a part of the study, this high defaulter rate would have been alarming for the IMPACT-TB study by introducing bias and uncertain diagnosis of a significant part of the children. However due to the intensive tracing of patients initially lost to follow-up, the final proportion of children with no follow-up data was only 5% (13/262). Non-adherence can impair the validity of data, be a significant financial burden for research project, and importantly be a threat to the health of the study participants [12]. It requires a large number of study participants to compensate for high drop out rates. Pledger et al. calculated that a 20% non-compliance rate would need 50% more subjects than if the study participants achieved 100% adherence [13]. But increasing the number of participants may not eliminate the risk of bias;

We found, in line with a review of HIV studies, Brinkhof et al. [14], that 25% of the children initially lost to follow-up, had died. In our study region it is possible that the children died from other diseases such as malaria and other severe infections but there is a considerable risk that that some actually died from TB. A high mortality with unknown cause of death, in a diagnostic study may bias and impair the validity of results, which cannot be compensated by increasing the number of study participants. Timely tracing of non-adherent patients would not only improve research results and validity but also improve the chances of preventing death by identifying children who are severely ill.

Most studies evaluating follow-up adherence have their main focus on adherence to treatment, such as adherence to ART (anti-retroviral therapy) and anti-TB treatment [15]–[16]. Very little has been published exclusively on adherence to follow-up in research projects, apart from adherence to Mantoux-test readings [17] and only a few studies describe the complications of patients lost to follow-up. Among four prospective studies from Europe, looking at QFT and diagnosis of paediatric TB, none described clinical follow-up examinations as part of their diagnostic criteria [18]–[21]. One Indian study discussed loss to follow-up but only in patients with discordant results [22]. Another study comparing the ELISPOT vs. TST for diagnosing active TB in children Liebeschuetz et al. [23] found that 26% of 262 patients were lost to follow-up, which lead to the fact that 44% of their study participants was grouped as ‘possible tuberculosis or lost to follow-up’.

Our findings are similar to findings of studies investigating adherence to treatment [24], but more studies are needed to confirm the findings in relation to the general issue of adherence to follow-up in research projects. Like other studies we found no association between adherence to follow-up, sex and age [25].

Receiving money for the bus fare was a significant positive predictor for attending 2-month follow-up and the interviews also revealed major concerns about money. Our findings are in line with the findings of a recent review of 44 qualitative studies investigating adherence to anti-TB treatment. This review found an association between adherence and financial costs and they found similar themes of financial burden, importance of family support, and communication [26]. Although most of these studies focused on adherence to treatment, similar constraints are seen in research settings.

At discharge, the caretakers received not only money for the bus fare but also a discharge card with the date of the 2-month follow-up appointment. Patients who did not receive money for the bus fare also did not receive the discharge card, which would have at least reminded caretakers of the follow-up date. All study participants were thoroughly informed about the study setup at inclusion but it was not possible for us to conclude whether it was the money, poor communication or the way patients were discharged that was the main cause of non-adherence. The qualitative interviews suggested that money was the major factor, but also that both good communication and the appointment card influenced adherence.

The positive correlation between being diagnosed with TB and follow-up adherence could be due to the DOTS (directly observed treatment short course) strategy for TB treatment. Patients receive the treatment at the TB clinic over a 6-month period and this approach seems to work [27]. The methods used in the DOTS program, to keep patients within the system, may be useful when considering strategies to ensure adherence within research.

Another reason for non-adherence could be that the child was healthy and the caretaker did not find it necessary to take the child to the hospital. In a Nigerian glaucoma study 50% of the patients stated the reason for dropping out as “feeling well” after the initial treatment, and travel problems [28]. We found that 77% of the defaulters were feeling well at the time they were traced in the villages, which could explain the non-adherence. However 41% of the defaulters had been readmitted to a hospital or health facility, between discharge and follow-up, which contradicts this “feeling well” hypothesis and the interviews underlined that caretakers were generally motivated to bring their children, even if the child was no longer ill.

Domestic factors influenced motivation for attending follow-up; It was usually the mother, who gave consent to enrol the child in the study, and our interviews revealed that it sometimes created problems when the husband was informed about the study. In a study investigating the quality of parental consent Pace et al. found that 94% of the caretakers decided themselves to enrol a child in a study and only 6% discussed this with their spouses. [29]. Thus to avoid this kind of problem primary caretakers should be encouraged to discuss the matter with their husbands before enrolling a child in a study.

Our study has some potential limitations; For the qualitative interviews of non-adherent children, patients were chosen to represent a diverse range of distances to the hospital, as well as diverse time of inclusion, whilst adherent patients were interviewed at the project facility. This may have resulted in a much shorter span of time in relation to inclusion in the adherent groups compared to the non-adherent group. But because of the qualitative approach it was considered to be a reasonable method for choosing the interviewed patients.

The time span from inclusion in the IMPACT-TB study to the clinical evaluation of the patients lost to 2-month follow-up in the villages varied from 3–17 months, therefore the clinical findings could not be directly compared to children who attended regular 2-month follow-up for the study. However, the purpose of follow-up in the IMPACT-TB study was to determine whether or not the child had TB and this purpose was achieved for all the children who were still alive at follow-up. For the children who died after discharge, we have no confirmed diagnosis. We are unable to conclude whether the death of 9% (21/262) of the total population may have affected our results.

Distance to the hospital may have been a factor affecting adherence and other studies have found an association between adherence to treatment and distance to the clinic [30]. We mapped the homes of all actively traced study participants with a GPS, in order to calculate the distance to the hospital. However since there were no GPS maps of the roads of Tanzania available at the time, it was impossible to correlate the actual travel distance to the hospital, due to the distribution and quality of roads, available transport and mountains.

Various strategies to overcome adherence problems have been suggested such as; having a proper strategy for defaulters and using multiple strategies to avoid patient dropout [31], education, familial support, provider interventions [32] and the use of a letter or a phone call [33]. Furthermore factors such as staff training, monitoring reports, data entry/management and participant contact are known to influence loss to follow-up [34]. In a resource limited setting though, it can be difficult to obtain useful contact information. Many patients do not have an address and few may have access to a phone. However cell phone networks are currently extending throughout many developing countries [35], and will be a promising tool in locating people, sending reminders and it will be possible to send credit as motivation to attend follow-up. Patient and staff education, clear explanations, encouragement, elimination of patient costs and improved recording of contact details should all be part of a broader strategy for ensuring good adherence to follow-up in a research study. Finally integrating research projects into other health services such as the DOTS program may ensure follow-up.

In conclusion we found that patients lost to follow-up was a potentially significant problem. Because active tracing of defaulters was part of the study set-up we ended up with a loss to follow-up rate of only 5%. Follow-up adherence in the IMPACT-TB study was negatively influenced by travel costs, poor communication at discharge and death, whereas receiving TB treatment in a DOT program positively influenced follow-up adherence. Careful study management is crucial to ensure that non-adherent patients are noticed early in the process and can be traced and seen as close to the original follow-up date as possible. Patient and staff education, clear explanations, encouragement, elimination of patient costs and ensuring that patients can be traced should all be part of a broader strategy for ensuring good adherence to follow-up in a research study.
